# Effects and mechanisms of action of sildenafil citrate in human chorionic arteries

**DOI:** 10.1186/1477-7827-7-34

**Published:** 2009-04-23

**Authors:** Chrisen H Maharaj, Daniel O'Toole, Tadhg Lynch, John Carney, James Jarman, Brendan D Higgins, John J Morrison, John G Laffey

**Affiliations:** 1 Department of Anaesthesia, University College Hospital, Galway, Ireland; 2 Department of Anaesthesia, Clinical Sciences Institute and National Centre for Biomedical Engineering Sciences, National University of Ireland, Galway, Ireland; 3 Department of Obstetrics and Gynaecology, Clinical Sciences Institute, National University of Ireland, Galway, Ireland

## Abstract

**Objectives:**

Sildenafil citrate, a specific phosphodiesterase-5 inhibitor, is increasingly used for pulmonary hypertension in pregnancy. Sildenafil is also emerging as a potential candidate for the treatment of intra-uterine growth retardation and for premature labor. Its effects in the feto-placental circulation are not known. Our objectives were to determine whether phosphodiesterase-5 is present in the human feto-placental circulation, and to characterize the effects and mechanisms of action of sildenafil citrate in this circulation.

**Study Design:**

Ex vivo human chorionic plate arterial rings were used in all experiments. The presence of phosphodiesterase-5 in the feto-placental circulation was determined by western blotting and immunohistochemical staining. In a subsequent series of pharmacologic studies, the effects of sildenafil citrate in pre-constricted chorionic plate arterial rings were determined. Additional studies examined the role of cGMP and nitric oxide in mediating the effects of sildenafil.

**Results:**

Phosphodiesterase-5 mRNA and protein was demonstrated in human chorionic plate arteries. Immunohistochemistry demonstrated phosphodiesterase-5 within the arterial muscle layer. Sildenafil citrate produced dose dependent vasodilatation at concentrations at and greater than 10 nM. Both the direct cGMP inhibitor methylene blue and the cGMP-dependent protein kinase inhibitor Rp-8-Br-PET-cGMPS significantly attenuated the vasodilation produced by sildenafil citrate. Inhibition of NO production with L-NAME did not attenuate the vasodilator effects of sildenafil. In contrast, sildenafil citrate significantly enhanced the vasodilation produced by the NO donor sodium nitroprusside.

**Conclusion:**

Phosphodiesterase-5 is present in the feto-placental circulation. Sildenafil citrate vasodilates the feto-placental circulation via a cGMP dependent mechanism involving increased responsiveness to NO.

## Background

Sildenafil citrate, a specific phosphodiesterase-5 inhibitor, has demonstrated considerable promise as a pulmonary vasodilator [1-4]. Sildenafil has been proposed as a potentially useful therapy for pulmonary hypertension in pregnancy, a disease characterized by poor maternal and fetal outcome [5,6]. Several case reports of its use in pregnant patients with pulmonary hypertension have been published to date [7,8]. The effects of sildenafil citrate on the pulmonary vasculature and on pulmonary artery pressure are increasingly well understood. Sildenafil citrate acts by reducing cGMP breakdown, making pulmonary vascular smooth muscle more sensitive to both endogenous and exogenous NO, reducing ventilation/perfusion mismatch and hypoxia [1,9,10].

Sildenafil is also emerging as a potential candidate for the treatment of intra-uterine growth retardation and for premature labor [11]. Sildenafil has also been proposed as a potential therapeutic strategy to maintain placental function in pre-eclampsia [12]. While placental transfer of sildenafil citrate has not been quantified, due to its chemical characteristics it is likely to easily cross the placenta into the fetus. Should sildenafil citrate possess vasodilatory effects in the feto-placental circulation, this would significantly enhance its therapeutic potential in the setting of placental insufficiency. Of interest, sildenafil citrate has recently been demonstrated not to alter the contractile response to vasoconstrictors or to endothelial dependent vasodilators[13]. However, the direct effects of sildenafil citrate in the feto-placental circulation have not been determined.

The purposes of these studies were to determine whether the phosphodiesterase-5 enzyme was present in the feto-placental circulation, and then to characterize the effects and mechanisms of action of sildenafil citrate in this circulation.

## Methods

Following approval by the Galway University Hospitals Clinical Research Ethical Committee, and written informed patient consent, term placentae were obtained following both vaginal and elective cesarean delivery under regional anesthesia from patients following normal pregnancy. None of the patients from whom the samples were taken received general anesthesia for delivery. Exclusion criteria included intra-uterine growth retardation, pre-eclampsia, and patients with pregnancy induced hypertension, hepatitis and HIV.

In all studies, umbilical arteries and their branches were identified as they spread out onto the chorionic plate of the placenta. Samples of the second-generation (second-order) chorionic plate arteries were taken within 120 minutes of delivery and placed directly into ice-cold pyrogen-free physiologic saline solution (122.6 mM NaCl, 5.4 mM KCl, 20 mM NaHCO_3_, 0.8 mM MgSO_4_, 0.9 mM Na_2_HPO_4_, 2.4 mM CaCl_2 _and 5.5 mM glucose) or flash frozen depending on the experimental requirements.

### Characterization of Phosphodiesterase-5

#### RT-PCR detection of Phosphodiesterase-5 mRNA

Freshly dissected chorionic plate arterial rings were homogenized in Tri-Reagent (Sigma Aldrich, Poole, Dorset, United Kingdom) using a TissueRuptor (Qiagen, Crawley, United Kingdom). RNA was then isolated as previously described [14]. RNA concentration was assessed using a ND-1000 Spectrophotometer (NanoDrop Technologies, Wilmington, DE USA), and 1 μg used to generate complementary DNA (cDNA) using an Access RT-PCR kit (Promega UK, Southampton, United Kingdom). Polymerase chain reaction was performed on the resulting cDNA in a DNA Engine thermal cycler (Bio-Rad Laboratories, Hercules, CA, USA). Detection primers used were:

Lamin A/C control: forward 5'-ATGGAGACCCCGTCCCAG-3'

reverse 5'-AGCTATCAGGTCACCCTCCTT-3'

PDE-5: forward 5'-TGGTCAATGCATGGTTTGCT-3'

reverse 5'-TCAGTCCATGGATATGCAAGA-3'

PCR products were analyzed on a 1% agarose (w/v) TAE gel and imaged using GeneSnap image acquisition software (Syngene, Cambridge, United Kingdom).

#### Western blot detection of Phosphodiesterase-5 Protein

Freshly dissected chorionic plate arterial rings were homogenized in ice-cold PBS, at 100 mg per mL, using a TissueRuptor (Qiagen). Samples were sonicated, and centrifuged at 14000 g for 5 minutes to remove insoluble proteins. SDS-PAGE sample buffer was added to various volumes of lysate and samples electrophoresed on a 4–20% acrylamide gel. Protein was transferred to nitrocellulose and probed using a mouse monoclonal PDE-5 antibody (Clone number H00008654-M01, Abnova, Walnut, CA, USA).

#### Immunohistochemical demonstration of Phosphodiesterase-5

Freshly dissected chorionic plate arterial rings were snap frozen in isopentane and embedded in OCT and sectioned to 15 μM thickness on glass slides as previously described [15]. Sections were permeabilized with 0.3% triton-PBS (v/v), and probed using PDE-5 primary (Abnova) and phycoeryhtrin labeled secondary (Sigma Aldrich) antibodies. Coverslips were sealed to the slides with Hardset DAPI (Vector Laboratories, Servion, Switzerland) mounting medium, and images taken under fluorescent microscopy. The coverslip was later removed and the section stained with haematoxylin and eosin to demonstrate ring structure.

### Effects and mechanism of action of sildenafil

**Figure 1:**
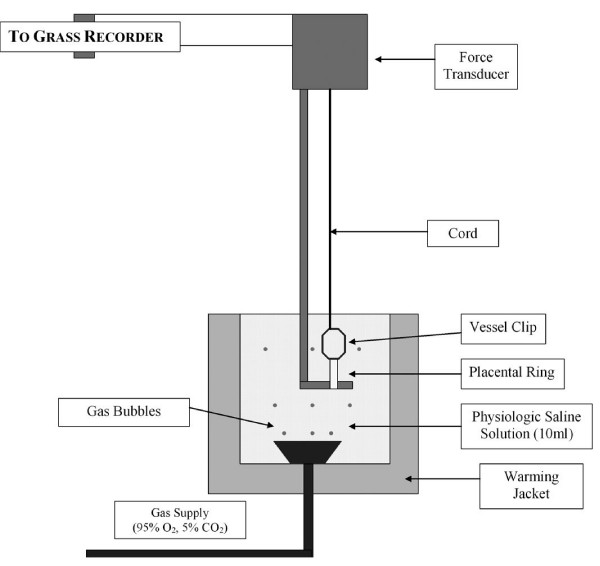
**Schematic representation of the ex vivo incubation model system used in the isolated vessel experiments**.

All studies, conducted in four separate series of experiments, followed a randomized, controlled, paired design. In each experiment, 4 chorionic rings, each 3 mm in length, from a single chorionic plate artery were isolated, mounted in physiologic saline solution at 37°C, and equilibrated with 95% O_2 _– 5% CO_2 _in tissue baths (10 ml capacity) as previously described [16,17] (Figure 1). Samples of the solution were intermittently analyzed for PO_2_, PCO_2 _and pH, using an automated blood-gas analyzer (ABL500, Radiometer Copenhagen, Denmark). Rings were threaded onto a horizontally fixed platinum surgical wire (150 μm diameter). A second hook, connected to an isometric force transducer, was then passed through the lumen of the ring (Figure 1). Isometric tension was recorded as a function of time using a transducer system (Grass FT03, Quincy, MA).

#### Baseline Interventions

Following a 60 minute equilibration period, an optimal pre-tension of 2.00 g for each chorionic plate arterial ring was utilized. These pretensions were determined in preliminary experiments in our laboratory to elicit active tensions of 80–100% of the maximum in each ring. Priming contractions were induced by exposing the rings three times to 80 mM KCl solution (iso-osmotically substituted for NaCl), and pre-tension re-established at the end of each five minute exposure by rinsing with physiologic saline solution. The paired preparations were then randomized to group allocation.

#### Experimental Design and Interventions

**Series 1 **examined the direct effects of sildenafil citrate on pre-constricted chorionic plate arterial rings. The rings were first submaximally pre-constricted with U46619 (9,11-dideoxy-11alpha, 9alpha-epoxymethanoprostagladin F2α, 5 × 10^-6 ^M), a thromboxane analog. This concentration was demonstrated in preliminary experiments to reliably produce a contraction of approximately 70% of the maximum amplitude obtainable. Once a stable plateau contractile response was obtained, the rings were allowed to remain at plateau for 30 minutes. Two rings from each placenta were then randomly assigned to receive sildenafil citrate or vehicle. A cumulative concentration response curve for sildenafil citrate (10^-10 ^to 10^-3 ^M) or vehicle was then constructed. The effects of sildenafil citrate were measured by calculating the mean amplitude of selected areas for the final 5 minutes of each 15 minute interval.

**Series 2 **examined the potential for direct inhibition of cGMP to attenuate sildenafil citrate induced vasorelaxation. Following pre-constriction with U46619, methylene blue (3 × 10^-5 ^M) a direct inhibitor of cGMP, was added to one sildenafil citrate exposed bath and one control bath, and vehicle added to the remaining baths. Following equilibration for 20 minutes, a cumulative concentration response curve for sildenafil citrate (10^-7 ^to 10^-3 ^M) or vehicle was constructed. This resulted in a four group design: (1) control with vehicle; (2) control with methylene blue; (3) sildenafil citrate with vehicle; (4) sildenafil citrate with methylene blue.

**Series 3 **examined the potential for direct inhibition of the cGMP-dependent protein kinase to attenuate sildenafil citrate induced vasorelaxation. In ***Series 3A***, following pre-constriction with U46619, two rings from each placenta were then randomly assigned to undergo pre-incubation with Rp-8-Br-PET-cGMPS (3 μM) or vehicle. A cumulative concentration response curve for sildenafil citrate (10^-7 ^to 10^-3 ^M) or vehicle was then constructed. This resulted in a four group design: (1) control with vehicle; (2) control with Rp-8-Br-PET-cGMPS; (3) sildenafil citrate with vehicle; (4) sildenafil citrate with Rp-8-Br-PET-cGMPS.

In ***Series 3B***, following pre-constriction, two rings from each placenta were then randomly assigned to undergo pre-incubation with Rp-8-Br-PET-cGMPS (30 μM) or vehicle. A cumulative concentration response curve for sildenafil citrate (10^-7 ^to 10^-3 ^M) or vehicle was then constructed. This resulted in a four group design: (1) control with vehicle; (2) control with Rp-8-Br-PET-cGMPS; (3) sildenafil citrate with vehicle; (4) sildenafil citrate with Rp-8-Br-PET-cGMPS.

***Series 4 ***examined the role of nitric oxide (NO) in mediating the relaxant effect of sildenafil citrate. The vasorelaxant effect of sildenafil citrate may be mediated via the generation of NO or via an increase in sensitivity to NO. ***Series 4A ***determined the potential for sildenafil citrate to produce vasorelaxation via NO generation. Two rings from each placenta were randomly assigned to undergo 20 minute pre-incubation with ω-nitro-L-arginine methyl ester (L-NAME, 1 × 10^-3 ^M), a non-specific inhibitor of Nitric Oxide synthase (NOS), or vehicle. A cumulative concentration response curve for sildenafil citrate (10^-7 ^to 10^-3 ^M) or vehicle was then constructed. This resulted in a four group design: (1) control; (2) L-NAME; (3) sildenafil citrate with vehicle; (4) sildenafil citrate with L-NAME.

***Series 4B ***examined the potential for sildenafil citrate to produce relaxation via an increase in sensitivity to NO. One ring from each placenta was randomly assigned to undergo pre-incubation with vehicle, sildenafil citrate (10^-8 ^M), and sildenafil citrate (10^-6 ^M) respectively, in a three group design. Following pre-constriction with U46619, sildenafil citrate (10^-8 ^M and 10^-6 ^M) or vehicle was then added to each bath and allowed to equilibrate for 20 minutes. A cumulative concentration response curve for sodium nitroprusside, an NO donor, in half log increments from 10^-9 ^to 10^-8 ^M was then constructed.

#### End Protocol Contractility Assessment

At the end of each experimental protocol, pre-tension was re-established by rinsing with physiologic saline solution. A recovery time of 30 minutes was then allowed. The contractile response to 80 mM KCl was then reassessed in order to assess performance over the course of the experiment. A final exclusion criterion was applied at this point, with data from all four rings excluded from analysis if the maximum final KCl-induced contraction in controls was less than 90% of the initial KCl response.

#### Chemicals

U46619, methylene blue, L-NAME, Rp-8-Br-PET-cGMPS, and sodium nitroprusside were purchased from Sigma Aldrich (Poole, Dorset, UK). All salts were purchased from Lennox Laboratory Supplies (Dublin, Ireland). The sildenafil citrate was obtained from Pfizer Limited, UK (Sandwich, Kent).

### Statistical Analysis

Data are presented as means ± SEM, with contractile response to U46619 expressed in grams and relaxation response to sildenafil citrate and sodium nitroprusside expressed as a percentage of baseline submaximal contraction. Comparison between control and test rings was made using two way repeated measures analysis of variance, with group and sildenafil/vehicle concentration as factors. Between group analyses were restricted to comparisons relevant to our *a priori *hypotheses, and were made using student's t testing with corrections for multiple comparisons. The null hypothesis was rejected for p < 0.05.

## Results

Chorionic plate arterial rings were obtained from placentae from 55 women ((median age, 26 years [range, 18–42 years]; median parity, 1 [range, 0–4]) following uncomplicated full term (median gestation 40 wks [range 38 – 41]) gestation, for these studies.

### Characterization of phosphodiesterase-5 in chorionic arteries

**Figure 2:**
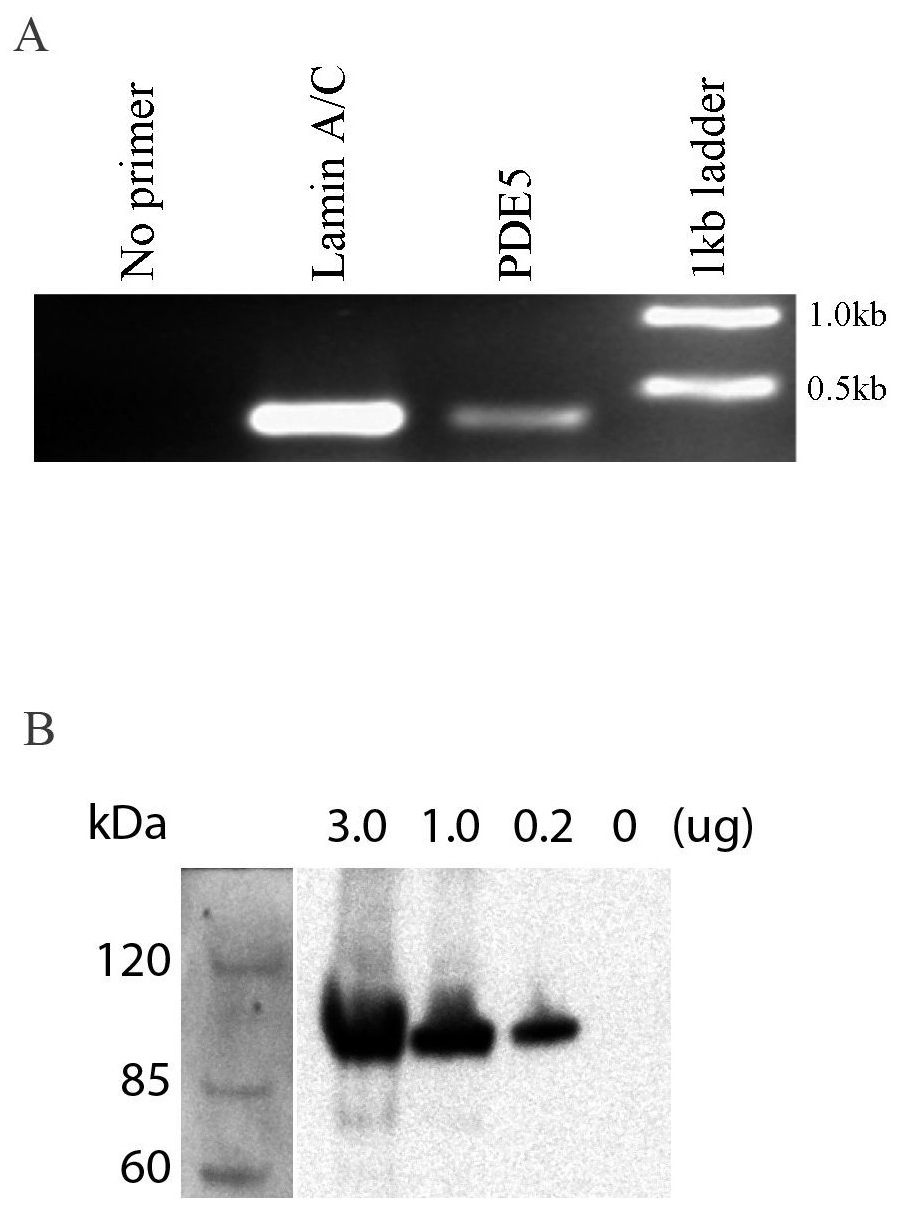
**Panel A: Demonstration of PDE-5 mRNA in chorionic plate arterial rings**. cDNA was prepared from RNA extracted from chorionic plate arterial rings, and amplified using specific primers for 400 bp regions of the indicated genes. PCR products were electrophoresed on 1% agarose. **Panel B:** Demonstration of PDE-5 protein in chorionic plate arterial rings. Human chorionic plate arterial rings homogenates, representing various amounts of protein, were examined by SDS-PAGE Western blot for the presence of PDE-5.

Phosphodiesterase-5 mRNA is present in chorionic plate arterial rings as demonstrated by RT-PCR. cDNA prepared from RNA extracted from homogenized chorionic plate arterial rings, and amplified by PCR using specific primers for the phosphodiesterase-5 gene, and electrophoresed on 1% agarose, demonstrated a band for phosphodiesterase-5 (Figure 2*** Panel A***).

The Phosphodiesterase-5 protein is present in chorionic plate arterial rings as demonstrated by Western blot. SDS-PAGE Western blot of protein samples from chorionic plate arterial ring homogenates were demonstrated to specifically bind PDE-5 antibody (Figure 2*** Panel B***).

**Figure 3:**
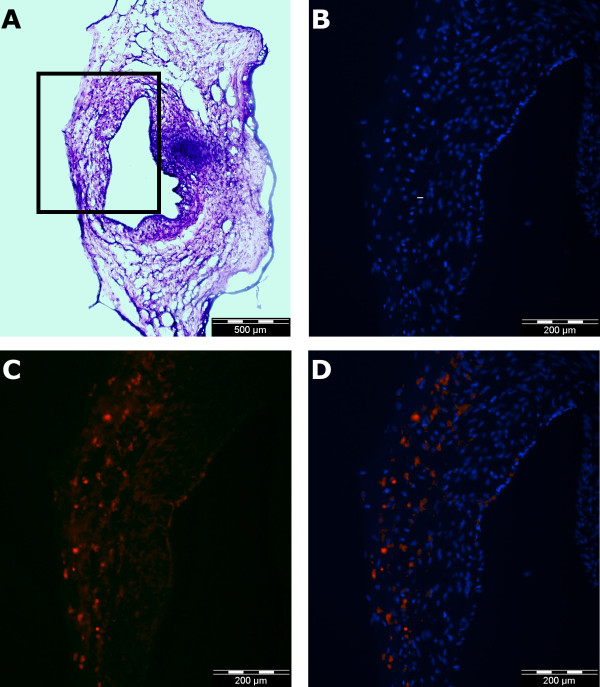
**Immunohistochemical demonstration of the localization of PDE-5 protein in chorionic plate arterial rings**. Haematoxylin and eosin staining of a paraffin embedded 15 μm section from a chorionic plate arterial ring demonstrates the structure of the ring (*Panel A*). Subsequent images, enlarged from the section of the wall of this ring indicated by the rectangle, demonstrate nuclear staining with DAPI (*Panel B*), staining with PDE-5 primary and phycoerythrin labeled secondary (Sigma) antibodies (*Panel C*), and the superimposition of panels B and C to demonstrate the cytoplasmic location of PDE-5 (*Panel D*).

Fluorescent microscopy of paraffin embedded slices of chorionic plate arterial rings demonstrated specific binding of PDE-5 primary antibodies within the cytoplasm of cells in the muscle layer within the arterial ring section. There is comparatively little staining for PDE-5 in the endothelial layer (Figure 3*** Panels A – D***).

### Sildenafil effects and mechanism of action

Stable and comparable gas tensions were maintained throughout all experiments, and comparable baseline levels of contractile responses were observed in all series. Post-intervention responses to potassium chloride were not different between the groups in any series.

#### Effects of sildenafil citrate on contractility of chorionic arterial rings

**Figure 4:**
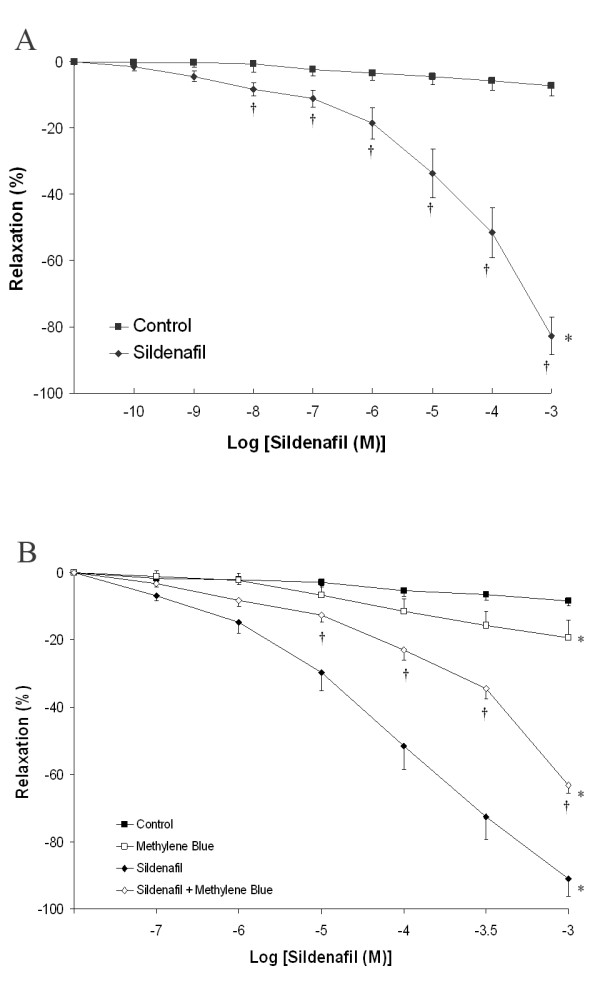
***Panel A*: Cumulative concentration-response curve for sildenafil citrate from 10^-10 ^M to 10^-3 ^M compared to control (vehicle) at a submaximal U46619 induced contraction**. *P < 0.05 compared to baseline. † P < 0.05 compared to control. ***Panel B*:** Cumulative concentration-response curve for sildenafil citrate from 10^-7 ^M to 10^-3 ^M compared to vehicle at a submaximal U46619 induced contraction, in the presence and absence of methylene blue. *P < 0.05 compared to baseline. † P < 0.05 compared to sildenafil citrate.

In ***Series 1***, sildenafil citrate dose dependently dilated chorionic arterial rings (n = 20 rings per group) following U46619 pre-constriction. Mean relaxation increased from 1.6 ± 1.2% at 10^-10 ^M, 11.1 ± 2.6% at 10^-7 ^M to 82.7 ± 5.5% at 10^-3 ^M (± SEM) sildenafil citrate (Figure 4*** Panel A***). The concentration of sildenafil citrate that produced a 50% relaxation (EC50_r_) was 1.6 ± 0.4 × 10^-4 ^M. Sildenafil citrate resulted in significant vasodilation over control conditions at concentrations at or greater than 1 × 10^-8 ^M.

#### Role of cGMP on sildenafil citrate-induced chorionic arterial ring vasorelaxation

**Series 2 **examined the potential for incubation with methylene blue (3 × 10^-5 ^M), a direct inhibitor of cGMP, to attenuate sildenafil citrate induced Vasorelaxation (n = 10 rings). There was minimal vasodilation in the rings exposed to control conditions or methylene blue alone. Methylene blue significantly attenuated the vasodilation produced by sildenafil citrate (Figure 4***Panel B***), and significantly increased the EC50_r _for sildenafil citrate (1.5 ± 0.5 versus 4.5 ± 0.4 × 10^-4 ^M, P < 0.01). These findings indicate a role for cGMP in mediating the vasodilation produced by sildenafil citrate.

**Figure 5:**
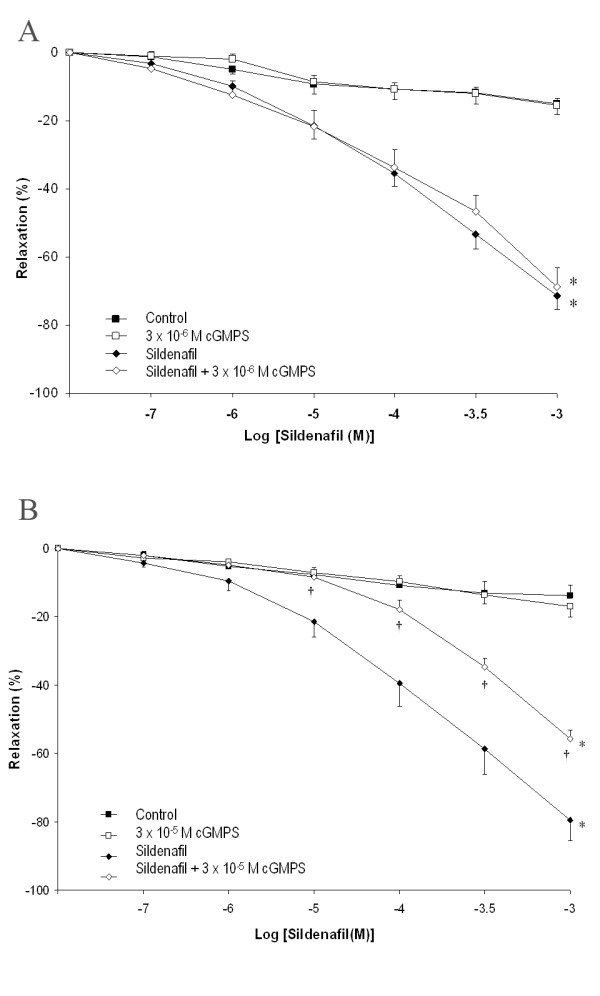
***Panel A*: Cumulative concentration-response curve for sildenafil citrate from 10^-7 ^M to 10^-3 ^M compared to vehicle at a submaximal U46619 induced contraction, in the presence and absence of cGMPS (3 × 10^-6 ^M)**. ***Panel B***: Cumulative concentration-response curve for sildenafil citrate from 10^-7 ^M to 10^-3 ^M compared to vehicle at a submaximal U46619 induced contraction, in the presence and absence of cGMPS (3 × 10^-5 ^M). *P < 0.05 compared to baseline. † P < 0.05 compared to sildenafil citrate.

**Series 3 **examined the potential for direct inhibition of the cGMP-dependent protein kinase to attenuate sildenafil citrate induced Vasorelaxation. In ***Series 3A***, there was minimal vasodilation in the rings exposed to control conditions or Rp-8-Br-PET-cGMPS (3 μM) alone (n = 10 rings). Rp-8-Br-PET-cGMPS (3 × 10^-6 ^M) did not modulate the vasodilation produced by sildenafil citrate (Figure 5*** Panel A***). In ***Series 3B***, again there was minimal vasodilation in the rings exposed to control conditions or Rp-8-Br-PET-cGMPS (3 × 10^-5 ^M) alone (n = 10 rings). However the higher concentration of Rp-8-Br-PET-cGMPS (3 × 10^-5 ^M) significantly attenuated the vasodilation produced by sildenafil citrate (Figure 5*** Panel B***) and increased the EC50_r _for sildenafil citrate (3.3 ± 0.6 versus 24.7 ± 15.0 × 10^-4 ^M, P < 0.01). These findings indicate that the cGMP dependent effect of sildenafil citrate in producing vasodilation in the feto-placental circulation is mediated via the cGMP-dependent protein kinase.

#### Role of nitric oxide on the sensitivity of chorionic arterial rings to the vasoactive effect of sildenafil citrate

**Figure 6:**
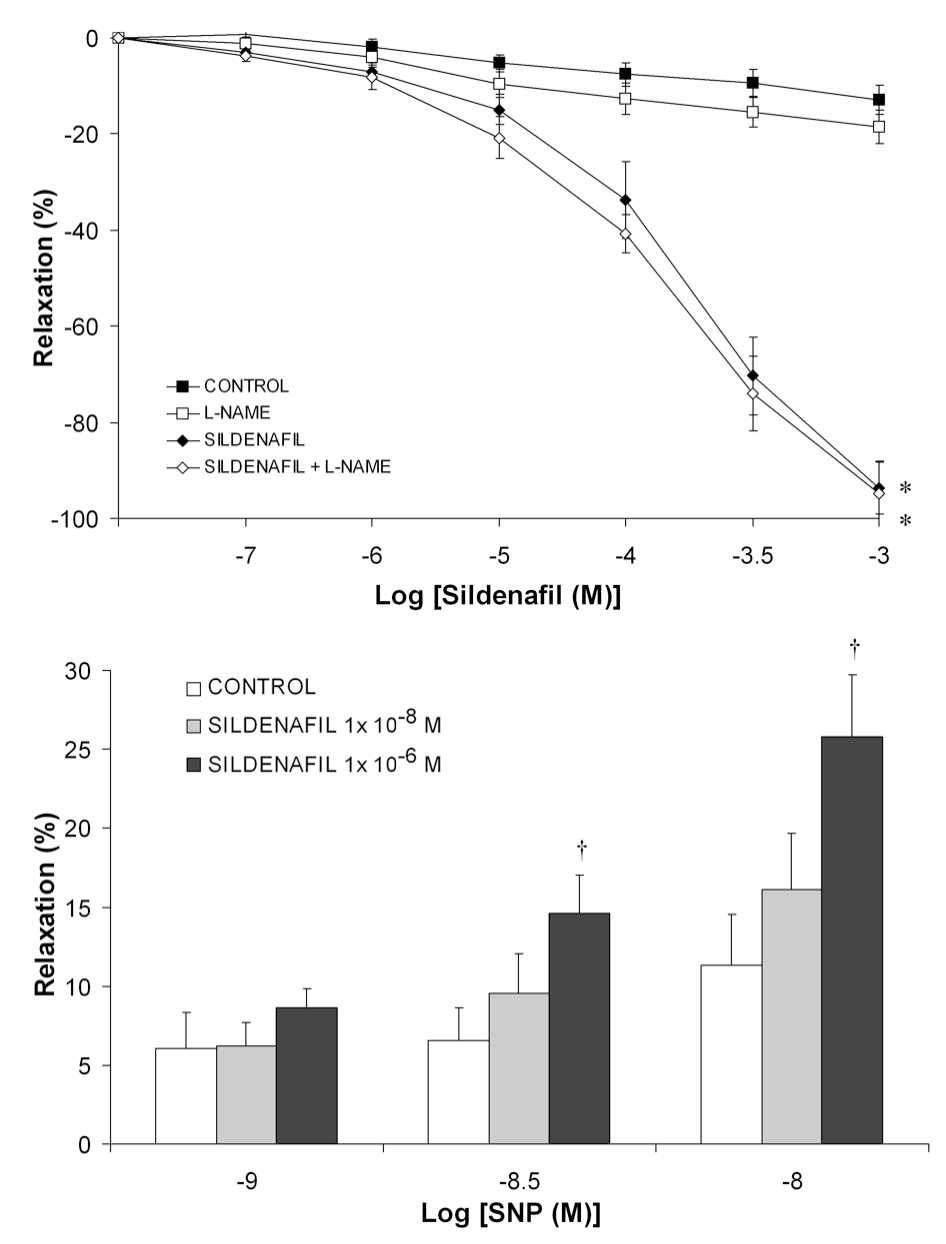
***Panel A*: Cumulative concentration-response curve for sildenafil citrate from 10^-7 ^M to 10^-3 ^M compared to vehicle at a submaximal U46619 induced contraction, in the presence and absence of L-NAME**. *P < 0.05 compared to baseline. ***Panel B***: Histogram of concentration dependent relaxation for sodium nitroprusside in the presence of increasing concentrations of sildenafil citrate. †P < 0.05 compared to control.

**Series 4A **examined the potential for incubation with L-NAME, a non-specific inhibitor of nitric oxide synthase, to attenuate sildenafil citrate induced Vasorelaxation (n = 10 rings). There was minimal vasodilation in the rings exposed to control conditions or L-NAME alone. L-NAME did not modulate the vasodilation produced by sildenafil citrate (Figure 6*** Panel A***).

**Series 4B **examined the potential for sildenafil to alter vessel sensitivity to exogenous NO (n = 10 rings). SNP dose dependently dilated pre-constricted rings, with a maximal relaxation of 11.3% ± 3.5% (mean ± SEM) at 10^-8 ^M SNP and 100% ± 4.2% at 10^-7 ^M SNP. At lower SNP concentrations (10^-9 ^to 10^-8 ^M), sildenafil citrate significantly, and dose dependently, increased the responsiveness of the rings to SNP (Figure 6***Panel B***).

## Discussion

Our results demonstrate the presence of phosphodiesterase-5 in the human feto-placental circulation and further demonstrate that sildenafil citrate is a vasodilator in this circulation. The vasodilatory effects of sildenafil citrate are mediated by via cGMP pathway, and involve an increase in vascular sensitivity to NO. These results are of clear significance given the growing role for sildenafil citrate for the treatment of pulmonary hypertension in pregnancy. In addition, these findings provide support for the hypothesis that sildenafil citrate may have a role in augmenting feto-placental blood flow in the setting of placental vascular insufficiency and pre-eclampsia [12].

Sildenafil citrate is increasingly used in the pregnant patient for the treatment of pulmonary hypertension in pregnancy, a disease associated with poor maternal [5] and fetal outcome [18]. Its safety and efficacy in this setting, combined with its lack of teratogenic or fetotoxic effects even at very high dosages in animal studies, mean that its use for the treatment of pulmonary hypertension in pregnancy is likely to increase [11]. Sildenafil citrate also causes relaxation of the human myometrium [19,20], and reduces intra-uterine pressures during preterm labor in rodents [21] and may therefore have a role as a tocolytic agent in the setting of premature labor. In addition, sildenafil citrate increases uteroplacental blood flow, and may have a role in the treatment of intra-uterine growth retardation. Sildenafil citrate improves the endothelial function of myometrial vessels from women whose pregnancies are complicated by intrauterine growth restriction [22]. Sildenafil enhanced fetal tolerability to induced intrapartum asphyxia, and increased fetal weight in guinea pigs [23]. Sildenafil increases rodent fetal size in the setting of hypoxia, induced by exposure of pregnant rats to environmental hypoxia, although this was not seen in the non-hypoxic setting [24].

The effects of sildenafil in the feto-placental circulation have not been characterized. In the uterine circulation, sildenafil citrate causes uterine artery vasodilation in non-pregnant females [25], and produces vasodilation in small myometrial arteries in women whose pregnancies are complicated by fetal growth retardation [22]. Should sildenafil citrate possess a similar vasodilatory effect in the feto-placental circulation, this would significantly enhance its therapeutic potential in the setting of placental insufficiency. It might also explain, in part, the beneficial effects seen on fetal growth in experimental studies of intrapartum asphyxia [23] and hypoxia [24]. While placental transfer of sildenafil citrate has not been quantified, due to its chemical characteristics it is likely to easily cross the placenta into the fetus.

Phospohdiesterase-5 has previously been demonstrated in the ovine feto-placental circulation [26]. In our study, we demonstrate the presence of mRNA and protein for phosphodiesterase-5, and confirm its presence immunohistochemically, in the human fetal placental circulation. This finding provided the rationale for our subsequent pharmacologic studies. These demonstrate that sildenafil citrate dose dependently vasodilates pre-constricted chorionic plate rings at concentrations of 1 × 10^-8 ^M and greater. Pharmacokinetic data suggest that a 100 mg oral dose of sildenafil citrate produces a plasma concentration in excess of 1 × 10^-7 ^M for 4 to 5 hours [27]. In addition, the peak plasma concentration achieved after an oral dose of sildenafil citrate 100 mg is 440 ng/ml, which equates to 1.5 × 10^-6 ^M [27]. Therefore, sildenafil citrate produced vasodilation in the feto-placental circulation at clinically relevant concentrations.

Our finding that Methylene blue, which is a direct inhibitor of cGMP, attenuated sildenafil citrate vasodilation, demonstrates that sildenafil citrate vasodilation is mediated via a cGMP dependent mechanism in this circulation. The direct cGMP dependant protein kinase inhibitor Rp-8-Br-PET-cGMPS (3 × 10^-5 ^M) significantly attenuated the vasodilation produced by sildenafil citrate, indicating that sildenafil citrate vasodilation is mediated via the cGMP-dependent protein kinase. Our finding that L-NAME did not inhibit sildenafil citrate vasodilation, indicates that *de novo *nitric oxide generation is not required to produce sildenafil citrate mediated vasorelaxation in this circulation. Conversely, sildenafil citrate potentiated the vasodilation produced by sodium nitroprusside, an exogenous NO donor, indicating that sildenafil citrate increased NO sensitivity.

There are a number of aspects of this study that indicate the need for caution prior to extrapolation to the clinical scenario. Firstly, these studies are conduced in chorionic plate arteries. Characterization of effects of sildenafil citrate on smaller vessels in the feto-placenta circulation (i.e. placental resistance arteries) is also required. Secondly, umbilical cord artery samples were obtained from healthy parturients. Characterization of the effects of sildenafil citrate in the setting of pulmonary hypertension of pregnancy, in which fetal hypoxia may be more frequent, would add further useful information. Finally, there are limitations in extrapolating from *in vitro *experiments to the *in vivo *situation, but the experiments conducted here represent a reliable and valid *in vitro *model for these vascular preparations.

In conclusion, our results demonstrate that phosphodiesterase-5 is present in the feto-placental circulation, and that sildenafil citrate is a vasodilator in this circulation. The vasodilatory effects of sildenafil citrate are mediated via a cGMP dependent mechanism, which involves in increase in vascular sensitivity to NO. These results are of clear significance given the growing role for sildenafil citrate for the treatment of pulmonary hypertension in pregnancy and may indicate a potential role for sildenafil citrate in augmenting feto-placental blood flow in the setting of placental vascular insufficiency.

## List of abbreviations used

U46619: 9,11-dideoxy-11alpha, 9alpha-epoxymethanoprostagladin F2; Rp-8-Br-PET-cGMPS: β-Phenyl-1, N^2^-etheno-8-bromoguanosine-3', 5'-cyclic monophosphorothioate, Rp-isomer; L-NAME: ω-nitro-L-arginine methyl ester.

## Competing interests

The authors declare that they have no competing interests.

## Authors' contributions

CM participated in the study design, performed the pharmacologic studies, and helped to draft the manuscript. DO'T performed the studies characterizing phosphodiesterase-5 and helped to draft the manuscript. TL conceived of the study, participated in its design and helped to draft the manuscript. JC, JJ and BH participated in the study execution, and helped to draft the manuscript. JM participated in the design of the study, and helped to draft the manuscript. JL designed and coordinated the studies, performed the statistical analysis, and helped to draft the manuscript. All authors read and approved the final manuscript.
